# Delabelling beta-lactam allergy

**DOI:** 10.3389/fphar.2024.1423719

**Published:** 2024-06-27

**Authors:** Gustavo-Jorge Molina-Molina, Diana Rocío Garnica Velandia, Blanca Andrés-López, Carolina Perales, Laura Marin-Asensio, Yanina Jurgens, Olga Esteso, Carolina Escobar, Xavier Vidal, Lourdes Vendrell, Laura Gómez-Ganda, Dolores Rodríguez, Eva Montané, Victoria Cardona, Antònia Agustí

**Affiliations:** ^1^ Allergy Department, Hospital Universitari de Bellvitge, Hospitalet de Llobregat, Spain; ^2^ Allergology Department, Hospital Universitari Santa Maria, Lleida, Spain; ^3^ Institut de Recerca Biomèdica de Lleida-Fundació Dr. Pifarré (IRB Lleida), Lleida, Spain; ^4^ Allergology Section, Hospital de Tortosa Verge de La Cinta, Institut D’Investigació Sanitaria Pere I Virgili, Tortosa, Spain; ^5^ Allergy Section, University Hospital Germans Trias I Pujol, Badalona, Spain; ^6^ Allergy Section, Hospital Universitari Joan XXIII de Tarragona, Tarragona, Spain; ^7^ Allergy Department, Hospital Universitari Dr. Josep Trueta, Girona, Spain; ^8^ Clinical Pharmacology Service, Hospital Universitari Vall D’Hebron, Barcelona, Spain; ^9^ Department of Pharmacology, Therapeutics and Toxicology, Universitat Autònoma de Barcelona, Barcelona, Spain; ^10^ Vall D’Hebron Institut de Recerca (VHIR), Barcelona, Spain; ^11^ Pharmacy Department, Hospital Universitari Vall D’Hebron, Barcelona, Spain; ^12^ Clinical Pharmacology Service, Hospital Universitari de Bellvitge, Hospitalet de Llobregat, Spain; ^13^ Clinical Pharmacology Service, University Hospital Germans Trias I Pujol, Badalona, Spain; ^14^ Department of Allergy, Hospital Universitari Vall D’Hebron, Barcelona, Spain

**Keywords:** beta-lactam, delabelling, drug provocation test, electronic medical records, skin test, penicillin

## Abstract

**Background:** Hypersensitivity to beta-lactam (BL) antibiotics is one of the most frequent reported drug allergies. In our population, it is common to find labels of BL allergy in electronic medical records (EMRs) that have not been assessed. The objective of our study was to detect patients with beta-lactam allergy labels in their EMRs and to assess how many of them are false after a correct diagnostic evaluation.

**Methods:** A multicentre prospective study was performed with patients labelled as allergic to BLs in their EMRs in the previous 5 years. Demographical and clinical data, as well as variables regarding the BL allergy label and the characteristics of the index reaction from clinical history and EMRs, were recorded. Then, diagnostic assessments including clinical history, skin tests (STs), and drug provocation tests (DPTs) were conducted in order to confirm or exclude the diagnosis of BL allergy.

**Results:** A total of 249 patients completed the study, of which 160 (64.3%) were women with a median age of 57 years (interquartile range [IQR], 45–68). The most frequent BL allergy labels detected were for penicillin (124), amoxicillin/clavulanic acid (61), and amoxicillin (54). Of the 204 patients who underwent STs, 20.1% were positive. DPTs were performed in 224 patients, showing good tolerance in 87.1% of cases. After the allergy diagnosis work-up, 186 patients (74.7%) were diagnosed as non-allergic to BL antibiotics.

**Conclusion:** In our study population, the number of patients labelled as allergic to BLs in their EMRs was similar to that in previously published studies, with proportions near to 75%–80% being falsely labelled as allergic to BLs.

## 1 Introduction

Up to 10% of the global population has been labelled as allergic to beta-lactam (BL) antibiotics, mainly penicillin (Trubiano et al., 2017). Despite this fact, in the majority of cases after an allergy assessment, labels can be removed in more than 70%–80% of cases in adults and more than 90% of cases in children (Doña et al., 2012; Zambonino et al., 2014). However, these percentages vary depending on the population and the study design. In hospitalized patients, the percentage of BL allergy labels range from 5% to 16% (van Dijk et al., 2016).

Generally, it is advisable to assess patients with BL allergy labels in order to confirm or rule them out, as well as to determine the alternative antibiotics for each patient (Mirakian et al., 2015; Marín et al., 2023). Overdiagnosis of BL allergy leads to use of alternative antibiotics. This translates into patients receiving broad-spectrum antibiotics that may be associated with increased rates of surgical site infections, antibiotic resistance, and higher rates of adverse effects and healthcare-associated infections (Blumenthal et al., 2016). Subsequently, this entails the need for longer hospitalizations, causing deleterious effects on the patient and community health and higher healthcare costs (Pépin et al., 2005; LeBlanc et al., 2006; Loo et al., 2006; Sastre et al., 2012; Macy and Ngor, 2013; Picard et al., 2013; Macy and Contreras, 2014; McDanel et al., 2015; Blumenthal et al., 2016; MacFadden et al., 2016; Wong et al., 2016; Blumenthal et al., 2018; Mattingly et al., 2018; Doña et al., 2022).

The *Institut Català de la Salut*, the main public healthcare provider in Catalonia, is responsible for 80% of primary care centres, with eight public hospitals under its commandment. Since 2008, it has adopted the use of electronic medical records (EMRs), and shortly after, the possibility of labelling drug allergies was enabled. Thus, every time the clinical history is consulted, it triggers a notification.

This study aimed to detect patients with BL allergy labels in their EMRs and to assess how many of them were false after a correct diagnostic evaluation. Furthermore, factors that could lead to a study of negative allergy were assessed. This information could provide us with local data on the true burden of BL allergy in order to take specific actions in a situation of increasing rates of development of multi-resistant bacteria.

## 2 Materials and methods

### 2.1 Study population and design

A multicentre and multidisciplinary prospective study to confirm or rule out BL allergy was performed between 2020 and 2022 in patients labelled during the previous 5 years as allergic to BLs in their EMR using a cohort from the *Institut Català de la Salut.*

Seven hospitals from the four districts of Catalonia were a part of the study.

### 2.2 Inclusion/exclusion criteria

Patients, aged 18–80 years, were included from a list of patients with at least one BL allergy label to a commercialized BL antibiotic in Spain. The list was extracted from the EMR in the SAP^®^ (IBM, United States) database system provided to the Area of Information System from the *Institut Català de la Salut.* In order to detect 20% of true BL allergic patients with a ± 5% precision, a sample of 250 patients with at least one BL allergy label was required. Thus, from the initial list, a randomized sample of patients for each centre was obtained, proportional to the number of BL allergy labels recorded in each hospital.

Patients not capable of providing their consent due to cognitive or physical impairment, as well as those in end-of-life situations or institutionalized, were excluded.

### 2.3 Data collection

The list of patients with BL allergy labels provided information about the date of the BL allergy label recording, the hospital where the recording was made, patient coding number, birthdate, and the beta-lactam antibiotic recorded.

Data on the demographic characteristics of patients, toxic habits, medical history (hypertension, diabetes, immunodeficiency, and other conditions that could require a frequent use of antibiotics), and atopic diseases were also collected.

Regarding the BL allergy reaction, the following information was recorded: the BL administered, route of administration, indication, number of tolerated doses before the reaction, date of the reaction, time interval between the last administration and the onset of the reaction, the type of reaction and its severity, duration until resolution, need of pharmacological treatment, and previous history of allergy to other BLs or other drugs. Moreover, information about the tolerance before and after the reaction to the BLs under study or to other antibiotics was also collected.

Antibiotics were classified using the Anatomical Therapeutic Chemical (ATC) classification system (ATC/DDD Index, 2019). To classify the adverse reactions and medical conditions, the dictionary of medical terminology MedDRA was used (MedDRA, 2022).

### 2.4 Procedures

Once the sample of patients was assigned to each hospital, researchers from the Clinical Pharmacology and Pharmacy services called the patients to explain the scope of the study. For those who accepted to participate and gave their oral consent, researchers reviewed their EMRs looking for information on the drug reaction: description of the reaction, information on the culprit BL, and information on the actions taken (indication to the patient, referral for study, etc.).

Subsequently, patients were visited by the allergists, who requested their written consent, reviewed the information, and carried out the clinical history and allergy work-up.

The severity of the original reaction was classified as mild (self-limited, no treatment or minimum treatment required), moderate (needs medical attention and required treatment/in-hospitalized patients do not extend hospitalization), severe (required hospital admission/in-hospitalized patients extend the hospitalization), or very severe (requires intensive treatment, life-threatening, and leaves medical sequels).

Patients were considered non-allergic when they had tolerated the culprit antibiotic after the original report of the reaction, without the need for further testing, or after a negative allergy work-up. These patients were also included in the study. Their EMRs were reviewed, and a thorough clinical history was obtained concerning the index reaction, ensuring their subsequent tolerance to the culprit BL.

Proven allergy was considered if patients had already undergone a correct confirmatory study or if they tested positive in the following diagnostic protocol (Bourke et al., 2015).

• Low-risk patients (non-immediate reactions, >1 h of latency, and limited to skin symptoms): A direct drug provocation test (DPT) was performed with the suspicious BL if known or otherwise with amoxicillin–clavulanic acid.
• Other patients: Skin tests (STs) were performed following previously published protocols, followed by a DPT guided by the ST results (Broyles et al., 2020).
• Patients with non-immediate reactions involving severe mucocutaneous symptoms or direct organ damage were not tested with STs or DPTs. In the case of severe anaphylaxis, the risk was assessed individually, and the study was adapted accordingly (dilution of STs, initiation of exposure tests at lower doses, establishing intravenous access, etc.).

Skin prick tests (SPTs) and intradermal tests (IDTs) were performed with benzylpenicilloyl-poly-L-lysine (PPL) and sodium benzylpenicillin (MD) (Diater Laboratory, Madrid), benzylpenicillin (SPT 10,000 U/mL; IDT 1,000 U/mL and 10,000 U/mL), amoxicillin (20 mg/mL), clavulanic acid (20 mg/mL), and cefuroxime (2 mg/mL). When indicated, other BL STs were performed using intravenous standard formulations. The results were read following standardised interpretation (Brockow et al., 2002).

DPTs were carried out as follows.

• With the culprit antibiotic, or with amoxicillin–clavulanic acid if the culprit was unknown, until a target therapeutic dose was reached if the SPTs/IDTs were negative.
• With penicillin V in patients with SPTs/IDTs selectively positive to amoxicillin or cefuroxime.
• With cefuroxime in patients with SPTs/IDTs positive to PPL or MD or in those who had a positive DPT to penicillin V or amoxicillin in order to rule out a broad-spectrum allergy to BL.

In the case of non-immediate reactions, the antibiotic dose was repeated at home for 3 days. If the history was highly suggestive of allergy, and the study result was negative, the investigator may repeat the study after 2–4 weeks. A DPT was considered positive when the patient experienced symptoms and signs consistent with an allergic reaction. Reactions were considered immediate if they occurred within 60 min after the antibiotic intake with clinical symptoms like urticaria, pruritus, angioedema, or anaphylaxis and non-immediate when they occurred after 60 min and up to several days after exposure to the antibiotic, leading to the onset of morbilliform/maculopapular or urticarial rashes.

### 2.5 Outcomes

The primary endpoint was to assess the proportion of false/true BL allergy labels. Secondary endpoints were to describe the demographic and clinical characteristics of the bearers of BL allergy labels, the most frequent BL reported as the cause of hypersensitivity reactions, as well as the most frequent antibiotic alternatives used in these patients. Moreover, we examined the characteristics of the index reaction, severity, and proportion of patients in which this information in EMRs allowed the classification of the reaction.

Furthermore, we assessed the proportion of patients who were diagnosed as allergic to BL using STs and/or after a DPT.

Finally, predictive factors of a negative allergy test were explored.

### 2.6 Statistical analysis

The descriptive results of the continuous variables are expressed by means of the median and the first (Q1) and third (Q3) quartiles. The categorical variables are described with their frequency and percentage.

The primary analysis is the estimate of the proportion of true positive results, with Wilson confidence limits (CLs) at 95%. For the comparisons of continuous variables between the positive and negative groups, the Mann–Whitney test was used; for comparisons of categorical variables, the chi-square was used, or the trend test was used when one of them was ordinal. To examine the association between positive skin and/or IDTs and predictive factors, a multivariable logistic regression analysis was performed, where the positivity of the tests is the dependent variable and the demographic variables of the patients, the characteristics of the allergic reaction presented, and the causative BLs are independent variables. The same variables were used to examine the association between the positivity of each DPT and potential predisposing factors. The odds ratio (OR) and the 95% CL were calculated. Statistical significance was considered when the *p*-value < 0.05. Statistical analysis was performed using SAS^®^ 9.4 (SAS Institute Inc., Cary, NC, United States).

In order to simplify the analysis, cases were grouped into four main categories (penicillins, cephalosporins, monobactams, and carbapenems).

## 3 Results

**FIGURE 1:**
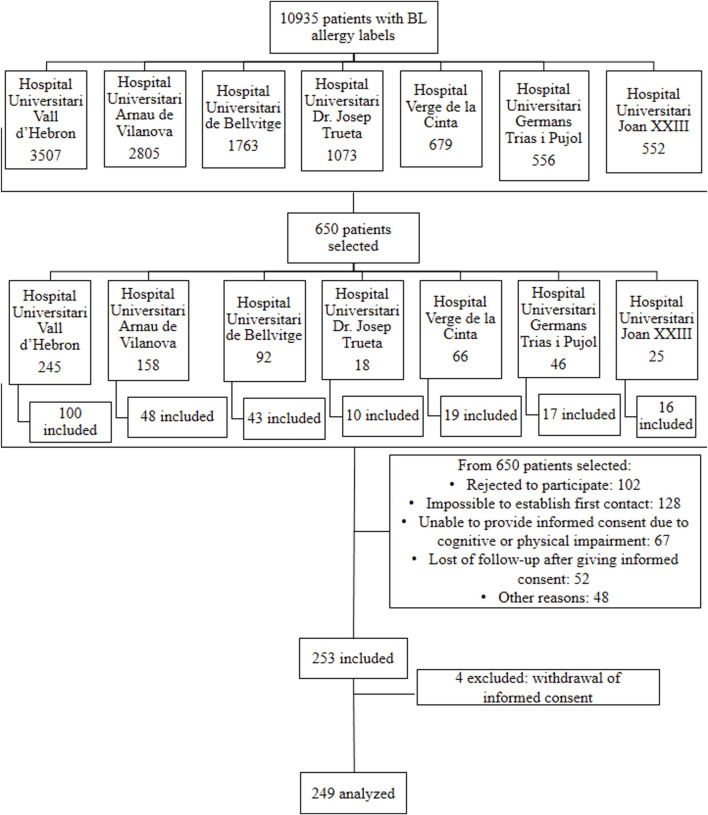
Flowchart of the distribution of patients labelled as allergic to BLs according to their reference hospital and the final inclusion in each centre.

In the previous 5 years, a total of 10,935 labels of BL allergy were recorded in the EMRs of patients aged 18–80 years from the seven hospitals participating in the study. As shown in Figure 1, 650 patients were randomly selected for the initial list and distributed proportionally based on the number of labels in each reference hospital. From this list, a sample of 253 patients was included in the study. A total of 397 patients were excluded, primarily due to the inability to establish initial contact to explain the study and because of refusal to participate. Four patients were excluded from the final analysis due to withdrawal of consent before the end of the procedures or due to refusal to be challenged to the suspected BL after negative STs. At that point, the recruitment phase of the study was closed. The final sample included 249 patients [women 64.3%, median age 57 years (interquartile range [IQR], 45–68).

The most frequent BL allergy labels detected in the EMRs were for penicillin (124), amoxicillin/clavulanic acid (61), and amoxicillin (54).

### 3.1 BL allergy delabelling

Of the 249 patients, 74.7% (186) were delabelled (95% CL, 68.9%–79.7%) after the allergy work-up. Of the 63 patients diagnosed as allergic to BLs, 54 had an immediate hypersensitivity reaction, and 9 had a non-immediate reaction.

Nine patients (3.61%) were delabelled through their clinical history as they had received the same BL after the index reaction without any reaction.

**FIGURE 2:**
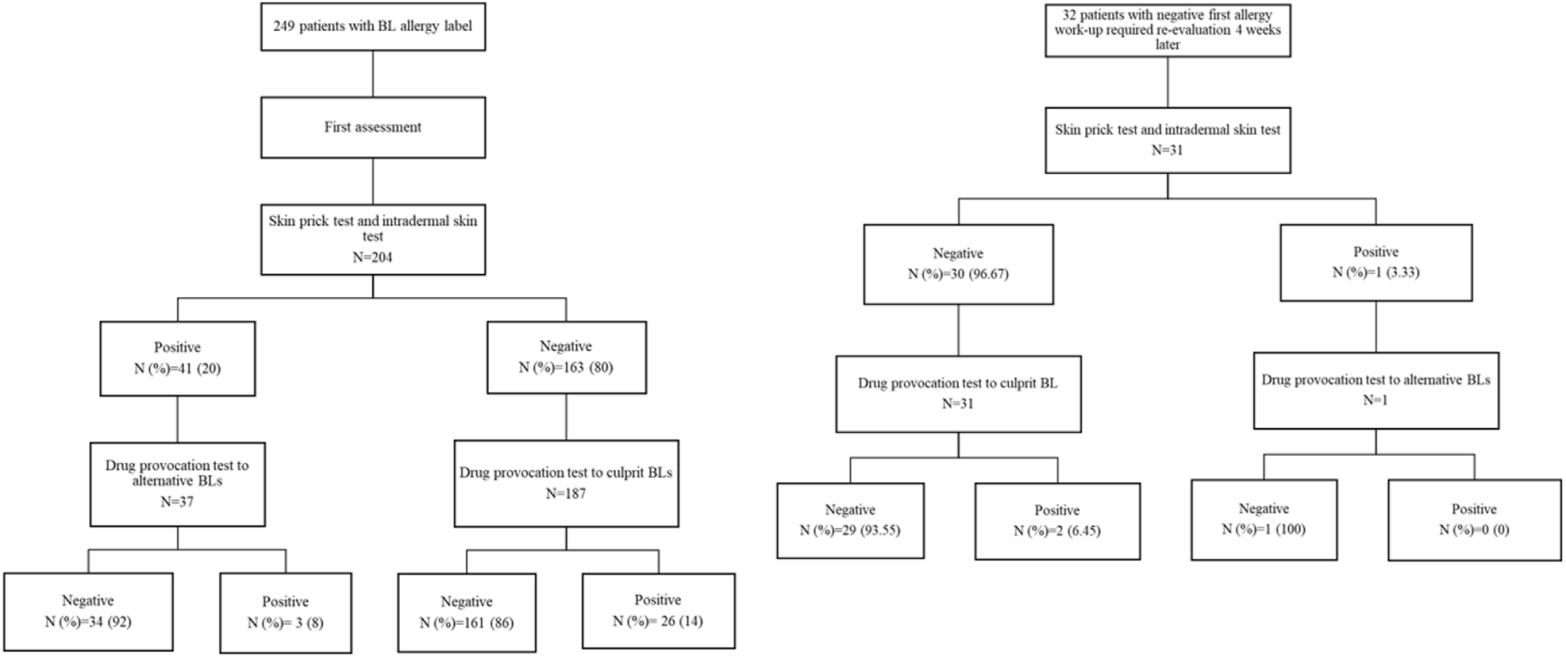
ST and DPT results of all patients after their first assessment and re-evaluation 4 weeks later.

Regarding the results of STs, DPTs, and re-evaluations, of the 204 patients who underwent STs, 41 (20.1%) tested positive. The DPT to the culprit drug was not carried out in the case of a positive ST. The distribution of DPTs to culprit drugs and alternative BLs is shown in Figure 2.

DPTs were performed in 224 patients, showing good tolerance in 195 (87.1%) of cases.

Re-evaluation after 4 weeks of the first assessment was required in 32 patients due to the high clinical suspicion of true BL allergy. STs were performed in 31 patients, with one positive case (3.33%). Of the 32 DPTs, two cases (6.67%) were positive (Figure 2). From these 32 patients, 25 had the index reaction for more than 2 years before their first allergy work-up. On the other hand, a total of 217 patients did not undergo re-evaluation due to low suspicion and non-severe reactions. Of these 217 patients, 169 cases had experienced the index reaction more than 2 years before their first allergy work-up. Regarding the 186 delabelled patients, 160 were not re-evaluated. Of those 160 patients, 125 experienced the reaction for more than 2 years before their first allergy assessment.

Table 1 presents the demographic and basal clinical characteristics of patients, showing no relevant statistical differences between the allergic and non-allergic groups, except for diabetes.

**TABLE 1:** Demographic and clinical characteristics of the patients with allergy labels to BLs.

	Total (*n* = 249)	Non-allergic (*n* = 186)	Allergic (*n* = 63)>	*p*
Age (median, IQR)	57 (45–68)	57.5 (44–70)	57 (46–64)	0.521
Sex	**–**	**–**	**–**	0.652
Women	160 (64.3)	121 (65.1)	39 (61.9)	**–**
Men	89 (35.7)	65 (34.9)	24 (38.1)	**–**
Medical history	**–**	**–**	**–**	**–**
Hypertension	91 (36.7)	72 (38.7)	19 (30.6)	0.254
Dyslipidemia	88 (35.3)	67 (36.0)	21 (33.3)	0.700
Diabetes	31 (12.5)	28 (15.1)	3 (4.8)	0.035
Asthma	25 (10.1)	17 (9.1)	8 (13.1)	0.372
COPD	22 (8.9)	17 (9.1)	5 (8.1)	0.797
Stroke	9 (3.6)	8 (4.3)	1 (1.6)	0.457
Coronary artery disease	22 (8.9)	20 (10.8)	2 (3.2)	0.071
Kidney failure	16 (6.5)	13 (7.0)	3 (4.8)	0.767
Immunodeficiency	16 (6.5)	9 (4.8)	7 (11.3)	0.130
Other antibiotic administration	15 (6.0)	12 (6.5)	3 (4.8)	0.768
Other conditions	104 (41.8)	77 (41.4)	27 (42.9)	0.839
Atopy history				
Familiar history	42 (17.4)	32 (17.5)	10 (17.2)	0.966
Atopic dermatitis	13 (5.2)	9 (4.8)	4 (6.5)	0.742
Allergic rhinitis	31 (12.6)	24 (13.0)	7 (11.1)	0.690
Allergic asthma	20 (8.1)	13 (7.1)	7 (11.1)	0.310
Food allergy	12 (4.8)	7 (3.8)	5 (7.9)	0.188
Chronic spontaneous urticaria	7 (2.8)	4 (2.2)	3 (4.8)	0.374
Other drug allergy	38 (15.3)	26 (14.0)	12 (19.0)	0.334

*BL, beta-lactam; IQR, interquartile range.

The highest percentages of delabelling according to the original labels in the EMR were for penicillin (91.1%), amoxicillin/clavulanic acid (63.9%), and amoxicillin (55.5%). It should be noted that in the cohort of patients finally diagnosed as allergic, the most frequent BLs that induced the index reaction were amoxicillin and amoxicillin/clavulanic acid. Regarding the 22 patients whose initial reactions were induced by amoxicillin/clavulanic acid, it was confirmed in 12 cases that the elicitor was clavulanic acid. The frequency of BL allergy labels according to the cohorts of allergic and non-allergic patients and the delabelling percentage are shown in Table 2.

**TABLE 2:** Distribution of the frequency of BL allergy labels detected according to the final diagnosis of drug allergy.

Suspected drug	Allergic *N* = 63 (%)	Non-allergic *N* = 186 (%)	% delabelled (95% CL)	Total *N* = 249
Amoxicillin	24 (38.09)	30 (16.15)	55.56 (42.38–68.00)	54
Amoxicillin/clavulanic acid	22 (34.92)	39 (20.96)	63.93 (51.39–74.83)	61
Cephalosporin	4 (6.34)	2 (1.07)	33.33 (9.68–70.00)	6
Penicillin	11 (17.46)	113 (60.75)	91.13 (84.81–94.97)	124
Unknown	2 (3.19)	2 (1.07)	50.00 (15.00–85.00)	4

*BL, beta-lactam; CL, confidence limit.

### 3.2 Severity of reactions

The severity of the initial reaction was stratified in 232 patients by the allergist through their clinical history, but was missing for 17. On the other hand, this information was available in the EMR in only 50 cases. The non-allergy cohort of patients reported mostly mild reactions, whereas truly allergic patients reported more severe reactions, as shown by the results of the significant trend test (*p* = 0.002) (Table 3).

**TABLE 3:** Severity of onset reactions according to non-allergic and allergic groups of patients.

Severity	Non-allergic *N* = 171 (% of non-allergic)	Allergic *N* = 61 (% allergic)	Total *N* = 232
Mild	109 (63.74)	25 (40.98)	134
Moderate	57 (33.33)	32 (52.46)	89
Severe	4 (2.34)	2 (3.28)	6
Very severe	1 (0.58)	2 (3.28)	232

### 3.3 Comparison between data available in EMRs and clinical history

There was a notable consistency between the BL allergy labels reported in the EMR and the clinical history in the majority of cases. Nevertheless, in 30 (12%) cases labelled as penicillin allergic, the patient was unable to recall the exact drug involved in the reaction, while in four cases, the drug recalled by the patient was a different one.

The date of the index reaction was not recorded in the EMRs in 178 (71.48%) cases, whereas by clinical history, six (2.4%) patients did not have their index reaction date recorded. In the EMR, information on the number of doses of BL ingested before the index reaction was absent in 207 (83.13%) of the cases and was classified as unknown in four cases. On the other hand, by clinical history, the aforementioned information was unknown in 109 cases, but registered in every case.

The time interval between the intake of BL and the onset of the index reaction was missing in the EMR in 210 cases (84.33%). By clinical history, this item was registered in every patient, despite it being unknown in 120 (48.19%) cases.

### 3.4 Age at the time of the reaction and time since the onset of symptoms

**FIGURE 3:**
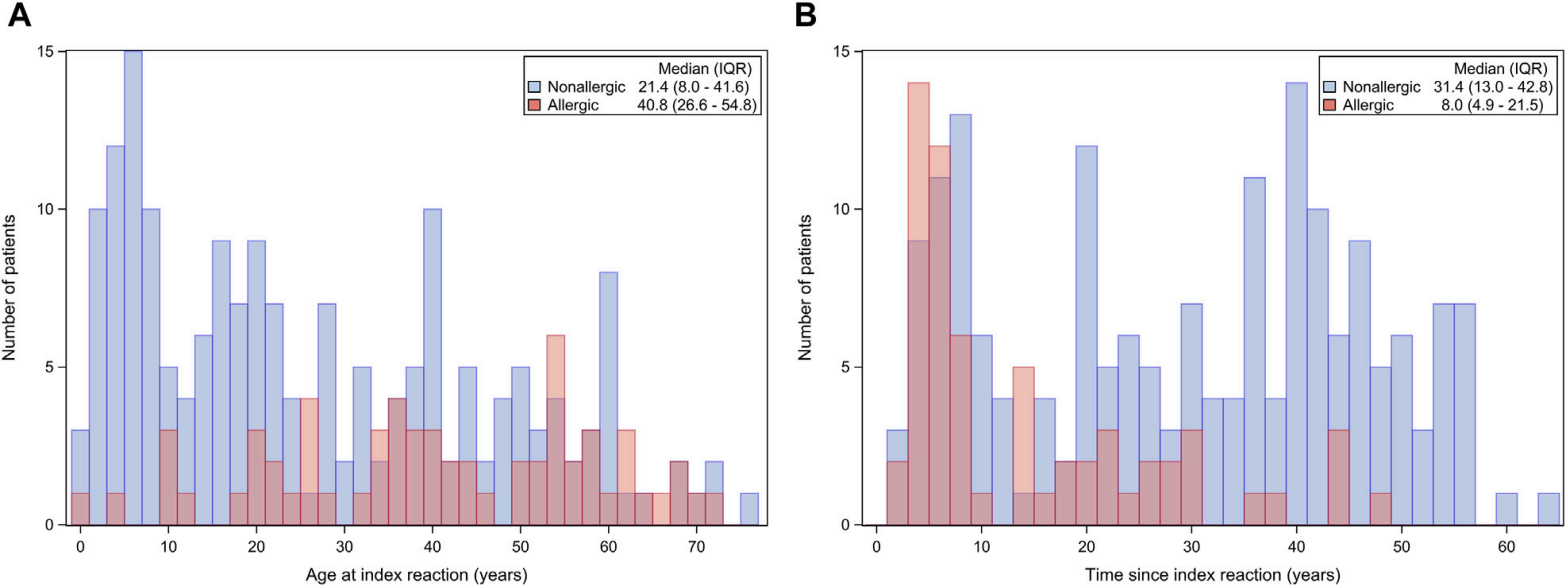
**(A)** Number of allergic patients according to the age at the time of the index reaction. **(B)** Number of allergic patients according to the time passed since the onset of the index reaction.

On one hand, as shown in Figure 3A, the number of allergic patients increases with age at the time of the reaction, showing the lowest proportions before the age of 20.

On the other hand, as shown in Figure 3B, the number of allergic patients tends to decrease with increase in the time since the index reaction. The highest proportion of allergic patients tends to aggregate in the first 10 years since the intake of the drug.

### 3.5 Predictive factors of positive/negative allergy tests

Regarding the predictive factors for positive or negative allergy tests, the OR of being allergic having an allergy label to amoxicillin was 7.79 (95% CL, 2.99–20.28) compared with being labelled allergic to penicillin (Table 4 **)**. In the case of being labelled allergic to cephalosporin, the OR was 44.97 (95% CL, 4.03–501.8) compared to being labelled allergic to penicillin.

**TABLE 4:** Estimation of the probability of being allergic comparing penicillin allergy labels to other BL allergy labels.

	OR (95% CL)
Drugs	
Penicillin	1 (reference)
Amoxicillin	7.79 (2.99–20.28)
Amoxicillin–clavulanic acid	5.83 (2.29–14.84)
Cephalosporins	44.97 (4.03–501.8)

*BL, beta-lactam; OR, odds ratio; CL, confidence limit.

**FIGURE 4:**
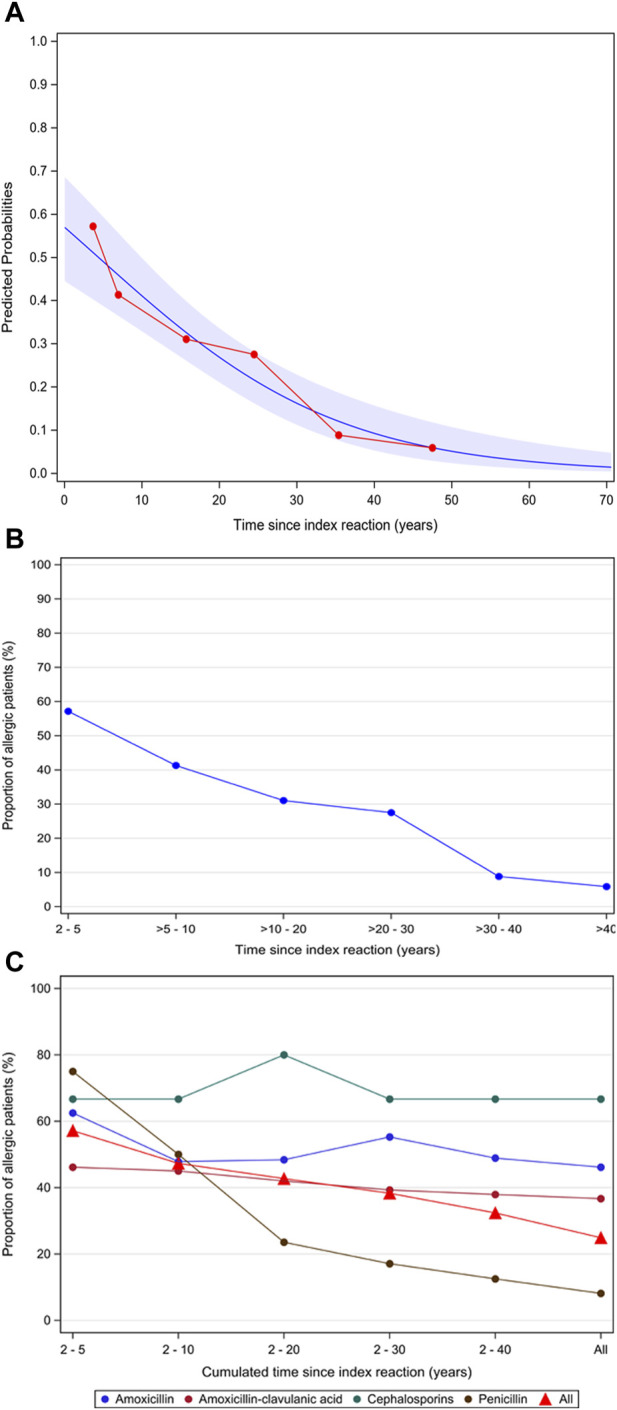
**(A)** Predicted probability of being diagnosed as allergic according to the time passed since the index reaction. **(B)** Reduction in the proportion of allergic patients according to the time passed since the index reaction. **(C)** Reduction in the proportion of allergic patients according to the culprit drug since the index reaction. Figure 4A: In blue, the probability predicted by the logistic regression model, which estimates the OR of 0.56, is represented, with the 95% confidence bands; in red, the observed values.

Figure 4A shows the reduction in the predicted probability of being diagnosed as a true allergic after each decade since the index reaction, with an estimated OR of 0.56 (95% CL, 0.39–0.80). Figure 4B shows the reduction in the proportion of patients diagnosed as allergic taking into account the time since the index reaction. Figures 4A,B are related due to the fact that Figure 4B is a modification of Figure 4A to represent the mean observed value over time in each interval with a decrease in the predicted probability of being allergic and the proportion of patients diagnosed as allergic by approximately 44%. It can be observed that the fit of values is adequate between both figures.

Differences in the reduction in the number of allergic patients according to drugs, especially with penicillin, are shown in Figure 4C.

Taking into account the severity of the original reaction in patients having suffered a non-mild hypersensitivity reaction (moderate to severe), the probability of being truly allergic was 3.42 (95% CL, 1.56–7.48) times higher (Table 5).

**TABLE 5:** Estimation of the probability of being allergic based on the severity of the initial reaction.

Severity	
Mild	1 (references)
Non-mild	3.42 (1.56–7.48)

Based on the symptoms of the allergic reaction, the presence of urticaria increased the probability of being allergic to BL antibiotics by 2.36 (95% CL, 1.06–5.24) times (Table 6).

**TABLE 6:** Estimation of the probability of being allergic based on the symptoms of the index reaction.

Symptom	
Urticaria	2.36 (1.06–5.24)
Angioedema	0.76 (0.30–1.91)
Syncope	1.39 (0.36–5.31)
Maculopapular exanthema	1.77 (0.68–4.63)

The multivariate analysis revealed that being labelled as allergic to amoxicillin, amoxicillin–clavulanic acid, and to cephalosporin increases the probability (raw OR) of being truly allergic by 3.33 (95% CL, 1.75–6.35), 2.10 (95% CL, 1.12–3.94), and 6.38 times (95% CL, 1.14–35.76), respectively, compared to being labelled as allergic to a different BL antibiotic (see Table 7).

**TABLE 7:** Multivariate analysis of the probability of true BL allergy.

	Raw OR (95% CL)	Adjusted OR (95% CL)
**Amoxicillin**	3.33 (1.75–6.35)	2.89 (1.37–6.11)
**Amoxicillin–clavulanic acid**	2.10 (1.12–3.94)	2.05 (0.99–4.25)
**Cephalosporin**	6.38 (1.14–35.76)	13.1 (1.29–133.5)
**Penicillin**	0.14 (0.07–0.28)	0.14 (0.06–0.32)

*BL, beta-lactam; OR, odds ratio; CL, confidence limit.

When the adjusted OR was calculated, taking into account the severity of the index reactions and the presence of urticaria, angioedema, syncope, or maculopapular exanthema, we observed that allergy labels to amoxicillin, amoxicillin with clavulanic acid, and cephalosporin played a role as risk factors for being truly allergic.

## 4 Discussion

We present a large multicentre prospective study in which the primary endpoint was to establish the true/false positive ratio of patients labelled allergic to BLs in their EMRs.

We found that the percentage of patients falsely labelled as allergic in their EMRs was 74.3%.

Although this is not the first epidemiological study that assesses the rate of false positive labels of BL allergy in EMRs, it is the first epidemiological study that provides data with a relevant representation of the different healthcare districts of Catalonia.

Other studies on this topic have been performed in Spain. In paediatric populations, the prevalence of true BL allergy was lower, ranging from 6% to 7.92% (Zambonino et al., 2014; Ferré-Ybarz et al., 2015; Sobrino et al., 2020). In adult patients, the results differ; while Moreno et al. (2016) reported in a large retrospective cohort that 28.6% of patients were allergic to BLs after the assessment, which is similar to the results of the current study, Ibáñez et al. (2018) showed that only 4.8% of 732 patients were finally confirmed to be allergic.

Studies in Spain are in line with those performed in other countries. In the United States, approximately 10% of all patients carry a label of penicillin allergy, yet less than 10% of these are confirmed after testing (Gadde et al., 1993; Trubiano et al., 2017; Blumenthal et al., 2019). Similarly, in Australia, Bourke et al. (2015) showed that 90% of patients labelled as allergic tolerated BLs. Abrams et al. (2016), in Canada, found an overall rate of false positive reports of 96.1% in their cohort of paediatric patients.

Regarding the basal characteristics of both allergic and non-allergic cohorts and their medical conditions, diabetes was the only item that showed statistical differences. One could hypothesize that diabetic patients, being at a higher risk of infections than the general population (Carey et al., 2018), might receive more antibiotics, including BLs. This increased antibiotic exposure could potentially elevate the risk of allergic reactions over time.

The most frequent BL allergy labels were for penicillin, amoxicillin/clavulanic acid, and amoxicillin. This is probably related to the frequency of use of BL antibiotics since it is consistent with the last report of the Spanish Agency for Medicines and Health Products, which reported in 2020 that the most frequent BLs used were amoxicillin alone or in combination with clavulanic acid (Agencia española del medicamento y de productos sanitarios, 2020). Since the EMR system allows the registration of an allergy alert to a specific antibiotic or a generic category like penicillin or BLs, many clinicians may choose wider categories in order to protect patients from accidental prescriptions. Thus, it is possible that the category of penicillin is being overestimated, a similar fact shown in previous studies (Ringwald et al., 2024). Furthermore, over the years, there may have been a trend change in the type of BLs used. Something similar occurs with the category “unknown.” It should be noted that in our EMR, it is not possible to label patients as allergic to clavulanic acid specifically, a fact that could lead to an underestimation of clavulanic acid hypersensitivity if not tested separately from amoxicillin. Even after a thorough clinical history, some patients are unable to recall the antibiotic they had received.

The lack of information registered regarding drug allergic reactions in the EMR is astonishing. Data on the specific BL that induced the index reaction are barely reported, preferring a label that encompasses the broader family of antibiotics. It is also important to note that the missing information about the date of the index reaction, the number of doses previously tolerated before the onset of the reaction, and the time interval since the last intake makes it impossible to classify the severity for adequate risk assessment. Therefore, completion of the information by means of a thorough clinical history is mandatory when assessing drug allergy.

The study shows the value of STs since they were positive in 20% of tested patients, while 12.9% of the challenges confirmed BL allergy. The rate of positivity in patients who underwent re-assessment was low (3.33% in STs and 6.66% in DPTs). Nevertheless, it stresses the relevance of re-evaluating patients with a highly probable clinical history.

These data were consistent with those in previous studies (Zambonino et al., 2014; Moreno et al., 2016; Solensky et al., 2019; Sousa-Pinto et al., 2021) that assessed the sensitivity and specificity of STs, finding a similar percentage of 30% of sensitivity and approximately 97% of specificity when they tested their patients during the allergy work-up. Our study began in 2020, and our allergy assessments were aligned with the position paper of the European Academy of Allergy and Clinical Immunology at that time (Romano et al., 2020). We determined that patients who had experienced severe immediate reactions to BLs more than 6 months prior to initial assessment and showed negative results in the allergy work-up should undergo re-testing.

Recent studies suggest that if the index reaction to BLs occurred more than 2 years ago and the initial assessment was negative, the cases should be re-evaluated, regardless of the severity of the index reaction (Doña et al., 2023; Freundt-Serpa et al., 2023). These studies found the highest rates of re-sensitization in patients who had moderate-to-severe reactions. In our study, the criterion of the occurrence of the index reaction more than 2 years ago was not considered when deciding whether to perform a re-assessment.

Multivariate analysis showed that the label in the EMRs to amoxicillin or cephalosporins, as well as non-mild severity of the index reaction, sustained by the OR and the tendency trend test, was associated with a true BL allergy. Furthermore, the presence or absence of urticaria and a short time since the last intake of a BL are variables that strongly affect the probability of being truly allergic. The strongest predictor of positivity was the time since the index reaction, showing reductions of 44% in positivity for every decade. There were differences in rate reduction in the proportions of allergic patients since the index reaction, taking into account the drug recorded in the allergy label. Penicillin showed the most notable rate reduction, followed by amoxicillin with clavulanic acid and amoxicillin alone. This may be related to the loss of hypersensitivity over time, as proposed by other researchers (Blanca et al., 1999; García Núñez et al., 2012; Romano et al., 2014; Torres et al., 2019). Regarding the relevance of specific antibiotic versus generic “penicillin” allergy labelling, we postulate that registering the specific name implies having a better source of information to check which BL was prescribed, better knowledge about the reaction, and probably a more recent event, in contrast with the tendency to use the generic label of penicillin allergy when information is scarce.

There are several limitations in this study. First, since this study was multicentric, and despite the study protocol being common for all, the individual criteria of the researchers could have resulted in some variations in the allergy assessment or the completion of the dataset. Recall biases of patients may have altered the description of the original reactions, which could have led to performing fewer re-assessments with the possibility of producing re-sensitizations to BLs even in small proportions. Furthermore, the study cohort was extracted from a public hospital database. Furthermore, as given in the Methods, we fell short by one patient to reach the chosen sample size of 250 patients, potentially impacting the statistical analysis power. Although unlikely, the results may not be applicable to patients managed by other healthcare providers or to the general population.

In conclusion, in our study population, the number of patients falsely labelled as allergic to BLs in their EMRs was similar to that in previously published studies, with proportions near 75%–80%. In a context of increasing global burden of bacterial antimicrobial resistance, proactive delabelling of false BL allergy is of major importance.

## Data Availability

The raw data supporting the conclusion of this article will be made available by the authors, without undue reservation.

## Abbreviations

ATC, anatomical therapeutic chemical; BL, beta-lactam; CL, confidence limit; MD, sodium benzylpenicillin; DPT, drug provocation test; EMR, electronic medical record; IDT, intradermal test; OR, odds ratio; PPL, benzylpenicilloyl-poly-L-lysine; ST, skin test; SPT, skin prick test.
